# Interaction Analysis of Abnormal Lipid Indices and Hypertension for Ischemic Stroke: A 10-Year Prospective Cohort Study

**DOI:** 10.3389/fcvm.2022.819274

**Published:** 2022-03-11

**Authors:** Lai Wei, Junxiang Sun, Hankun Xie, Qian Zhuang, Pengfei Wei, Xianghai Zhao, Yanchun Chen, Jiayi Dong, Mengxia Li, Changying Chen, Song Yang, Chong Shen

**Affiliations:** ^1^Department of Epidemiology, School of Public Health, Nanjing Medical University, Nanjing, China; ^2^Department of Cardiology, Affiliated Yixing People's Hospital of Jiangsu University, People's Hospital of Yixing City, Yixing, China

**Keywords:** lipids, hypertension, ischemic stroke, interaction, cohort study, dyslipidemia

## Abstract

**Background:**

Dyslipidemia and hypertension are two important independent risk factors for ischemic stroke (IS); however, their combined effect on IS remains uncertain.

**Objectives:**

This present study aimed to evaluate the interaction effect of hypertension and abnormal lipid indices on IS in a 10-year prospective cohort in Chinese adults.

**Methods:**

The cohort study of 4,128 participants was conducted in May 2009 and was followed up to July 2020. All qualified participants received a questionnaire survey, physical examination, and blood sample detection. Cox regression was used to evaluate the association of dyslipidemia and hypertension with IS, and calculate the hazard ratio (HR) and 95% confidence interval (CI). The relative excess risk of interaction (RERI) and the HR (95%CI) of interaction terms were used to examine additive and multiplicative interactions.

**Results:**

In the hypertensive population, Non-HDL-C ≥190 mg/dl, LDL-C/HDL-C ≥2 and HDL-C ≥60 mg/dl were statistically associated with IS, and after adjusting for covariates, HRs (95%CIs) were 1.565 (1.007–2.429), 1.414 (1.034–1.933) and 0.665 (0.450–0.983), respectively. While in the non-hypertension population, no significant association of Non-HDL-C ≥190 mg/dl, LDL-C/HDL-C ≥2, and HDL-C ≥60 was detected with IS (*P* > 0.05). There was a significant association between TC/HDL-C ≥ 3.6 and the decreased risk of IS in the non-hypertension population, and the HR (95%CI) was 0.479 (0.307–0.750). Whereas, a similar association was not observed in the hypertensive population. HDL-C ≥ 60 mg/dl, Non-HDL-C ≥ 190 mg/dl, TC/HDL-C ≥ 3.6, and TG/HDL-C ≥ 1 have additive and multiplicative interactions with hypertension (*P* < 0.05). The RERIs (95% CIs) of the additive interaction are −0.93 (−1.882–0.044), 1.394 (0.38–2.407), 0.752 (0.354–1.151) and 0.575 (0.086–1.065), respectively. The HRs (95% CIs) of the multiplicative interaction terms were 0.498 (0.272–0.911), 4.218 (1.230–14.464), 2.423 (1.437–4.086) and 1.701 (1.016–2.848), respectively.

**Conclusion:**

High concentration of HDL-C reduces the impact of hypertension on IS, while the high concentration of Non-HDL-C, TC/HDL-C, and TG/HDL-C positively interact with hypertension affecting the incidence of IS. This study provides useful evidence for the combined effects of dyslipidemia and hypertension in predicting IS.

## Introduction

Stroke is a major public health issue worldwide, with a high incidence rate, recurrence rate, disability rate, and mortality rate in the population (1). The global lifetime stroke risk from 25 years onward was estimated to be 24.9%, with China having the highest risk of stroke (39.3%), in 2016 (2). In China, stroke has also caused the highest number of disability-adjusted life years of all diseases and has been ranked third among the leading causes of death after malignant tumors and heart disease (3, 4). Moreover, due to changes in lifestyle and population structure and insufficient control over major risk factors, the burden it has brought to public health will be worsened in the future (5). Ischemic stroke (IS), the main subtype of stroke, accounts for almost 78% of stroke cases in China and takes up most of the health burden that stroke caused (6). IS usually occurs suddenly with acute signs and symptoms, which calls for emergency treatment. If emergency care is not provided timely, it will cause severe damage to blood vessels and nerves in the brain, resulting in irreversible complications, lifelong disabilities, or even death. However, the existing treatment for IS requires a strict time window, and the effect of its treatments is not satisfactory. The function damage to the brain will basically not recover fully after the stroke. Consequent side effects and a high risk of recurrence will continuously impact the prognosis and survivors' quality of life. Thus, it is key to focus on the management of risk factors to prevent IS.

IS is a multi-factorial disorder (7) of which prevention may require an improved understanding of modifiable risk factors. Several risk factors increase the IS incidence, such as hypertension, diabetes mellitus, hyperlipidemia, obesity, smoking, drinking, physical inactivity, and a family history of stroke (8, 9). Although each risk factor may contribute significantly to the development of IS, its occurrence is the result of a combination of multiple risk factors (10). According to GBD, over 90% of the global stroke burden is caused by the combined impact of modifiable risk factors (11).

Numerous studies have demonstrated that hypertension and dyslipidemia are two important independent risk factors that can be controlled and modified to prevent IS (12–15). Dyslipidemia is generally believed to play a critical role in the pathogenesis of IS, which can be represented by abnormal changes of traditional and non-traditional lipid indices. Traditional lipid indices, usually referring to total cholesterol (TC), triglycerides (TGs), low-density lipoprotein cholesterol (LDL-C), and high-density lipoprotein cholesterol (HDL-C), were identified to have predictive effects on the risk of cardiovascular disease and stroke (16–18). While compared with the traditional lipid indices, non-traditional lipid indices (non-HDL-C, TG/HDL-C, TC/HDL-C, and LDL-C/HDL-C) could serve as a more powerful predictor for vascular risk in stroke and CVD (19). Hypertension has a high co-occurrence rate with dyslipidemia, especially in the middle-aged and elderly population (20). A large cohort study in a French population showed a significantly higher risk of CVD in people under 55 years of age who suffered from comorbid hypertension and dyslipidemia (21). Therefore, exploring the possible interaction effect between hypertension and dyslipidemia on IS is of great significance for preventing and treating IS. However, few studies have examined the interaction of these two risk factors regarding IS. This present study aimed to evaluate the interaction effect of hypertension and abnormal lipid indices on IS in a 10-year prospective cohort in Chinese adults.

## Methods

### Study Design and Ethics Approval

This study adopted a prospective cohort design and a total of 4,128 individuals over 18 years old were recruited by a cluster sampling approach from 6 villages for baseline investigation in Guanlin Town and Xushe Town, Yixing City, Jiangsu Province from May to October 2009. The first field follow-up survey proceeded from May to October in 2014. After excluding 30 baseline stroke patients, the remaining 4,098 patients were followed up to July 27th, 2020 for stroke onset. For more information of this cohort, please refer to our previous published literature (22).

The Ethics Committee approved this study of Nanjing Medical University (#200803307). All participants or their caregivers provided signed informed consent before being included in this study.

### Data Collection and Related Covariates Definition

Population baseline investigation included questionnaire survey, physical examination, blood sample collection, etc. All the personnel involved in on-site investigations have received standardized training, and only after passing the assessment could they start their investigation.

Participants' demographic characteristics, smoking status, drinking status, and disease history were obtained from a validated questionnaire at the time of enrollment. Height, weight, and blood pressure were measured by standardized instruments, which were performed in duplicates to reduce random errors. Smoking was defined as cigarettes consumption greater than ≥ cigarettes per week, lasting at least 3 months a year. Drinking was defined as alcohol consumption ≥2 times per week, lasting at least 6 months per year. Body mass index (BMI) was obtained by dividing weight (kg) by the square of height (m^2^). Hypertension was defined as average systolic blood pressure (SBP) ≥ 140 mmHg or diastolic blood pressure (DBP) ≥ 90 mmHg, or currently receiving antihypertensive medication to lower blood pressure. The blood pressure of all subjects was measured 3 times with an interval of 30 s. If the difference between any two systolic or diastolic blood pressures is more than 8 mmHg, the fourth measurement is performed. Diabetes was defined as fasting plasma glucose (FPG) ≥7.0 mmol/l or a self-reported diabetes history. Received lipid-lowering treatment was defined as patients self-reported taking lipid-lowering medications or other lipid-lowering treatment measures.

All participants underwent 8-h overnight fasting and blood sampling to detect FPG and lipid indices, including total cholesterol (TC), triglycerides (TG), high-density lipoprotein cholesterol (HDL-C), and low-density lipoprotein cholesterol (LDL-C). Non-HDL-C, TC/HDL-C, TG/HDL-C, LDL-C/HDL-C, and remnant-cholesterol (RC) were derived from detected lipids. The non-HDL-C value was calculated as the difference value of TC minus HDL-C. RC was calculated by TC minus LDL-C minus HDL-C. Baseline dyslipidemia was defined by meeting any of the following conditions: (1) TC ≥ 6.2 mmol/l (240 mg/dl), TG ≥ 2.3 mmol/l (200 mg/dl), LDL-C ≥ 4.1 mmol/l (160 mg/dl), HDL-C < 1.04 mmol/l (40 mg/dl) or HDL-C ≥ 1.55 mmol/l (60 mg/dl); (2) Self-reported diagnosis of dyslipidemia; (3) Currently taking lipid-lowering drugs. As per Chinese Guidelines for the Management of Dyslipidemia in Adults (2016), the cut-off points of TC, TG, LDL-C, HDL-C, and non-HDL-C were 240 mg/dl, 200 mg/dl, 160 mg/dl, 40–60 mg/dl, and 190 mg/dl, respectively. While for indices like RC, TC/HDL-C, TG/HDL-C, and LDL-C/HDL-C, there are no definite clinical diagnostic criteria, so the median of these indicators, which were 30 mg/dl, 3.6, 1, and 2, were defined as the cut-off point value in this study.

### Outcome Ascertainment

Outcome events of stroke in this cohort were collected through the local register system of disease and death of the Center for Disease Control and Prevention (CDC). International Classification of Diseases, Tenth Revision, Clinical Modification (ICD-10-CM) was used to identify for stroke (I60~I64), IS (I63), and hemorrhagic stroke (I60, I61, I62, and I64). All the monitored stroke onset events were further inspected by certified neurologists and cardiologists through reviewing the medical records system and relevant files of Yixing People's Hospital.

### Statistical Analysis

Mann-Whitney *U* test was used to examine the differences of all the quantitative variables among groups of dyslipidemia presented as median (interquartile range). Chi-square (χ^2^) test was performed to compare the frequency distributions of qualitative variables. Cox proportional hazards regression models were used to estimate the HRs and 95% confidence intervals (CIs) of IS after adjustment for age, gender, smoking, drinking, BMI, hypertension, diabetes, and received lipid-lowering treatment. Heterogeneity was tested for inter-subgroup associations using Cochran's Q test. The interaction effect was evaluated using the relative excess risk of interaction (RERI) for additive interactions and the HR (95% CI) of multi-factor interaction terms for multiplicative interactions. All statistical analyses were conducted using SAS software version 9.4 (SAS Institute, Inc, Cary, NC), and test results were considered significant at the two-sided 0.05 level.

## Results

### Baseline Characteristics of the Cohort Study Population

Descriptive characteristics of the 4,098 participants at baseline are presented in Table 1. The participants were followed up for a median duration of 10.76 years, with 272 participants who developed IS. All participants were categorized into four groups according to the presence of dyslipidemia and hypertension at baseline. The proportion of people with only dyslipidemia, hypertension, and co-morbidity were 26.2, 20.8, and 27.6%, respectively. The median age is 59.20 years, with females accounting for 59.5% of the total population. Smokers and drinkers accounted for 24.3 and 21.5%, respectively. The prevalence of dyslipidemia, hypertension, and diabetes in the population was 53.9, 48.4, and 11.2%, respectively, at baseline. Participants with dyslipidemia or hypertension were more likely to have higher levels of BMI and higher proportions of drinkers and diabetes than their counterparts without dyslipidemia and hypertension (*P* < 0.05). The sex ratio and the proportion of smokers were approximately the same among four groups (Table 1).

**Table 1:** Baseline characteristics of the study population.

**Characteristics**	**All subjects**	**Non-dyslipidemia and non-hypertension**	**Only dyslipidemia**	**Only hypertension**	**Dyslipidemia and hypertension**	**Z/χ^2^**	***p*** **^a^**
	**(*N* = 4,098)**	**(*N* = 1,038)**	**(*N* = 1,075)**	**(*N* = 853)**	**(*N* = 1,132)**		
Age (years)	59.20 (52.75, 67.00)	56.78 (49.78, 63.81)	57.80 (51.77, 64.78)	61.75 (54.76, 69.77)	61.62 (54.74, 69.39)	144.538	<0.001
Gender (%)						2.541	0.468
Male	1,661 (40.5)	408 (39.3)	444 (41.3)	334 (39.2)	475 (42.0)		
Female	2,437 (59.5)	630 (60.7)	631 (58.7)	519 (60.8)	657 (58.0)		
Smokers (%)	995 (24.3)	258 (24.9)	266 (24.7)	191 (22.4)	280 (24.7)	2.095	0.553
Drinkers (%)	883 (21.5)	212 (20.4)	256 (23.8)	157 (18.4)	258 (22.8)	10.059	0.018
Diabetes (%)	461 (11.2)	80 (7.7)	112 (10.4)	86 (10.1)	183 (16.2)	42.362	<0.001
BMI (kg/m^2^)	24.00 (21.93, 26.40)	23.35 (21.51, 25.71)	23.50 (21.47, 26.02)	24.30 (22.33, 26.80)	24.88 (22.37, 27.28)	99.665	<0.001
TC (mg/dl)	185.33 (162.93, 210.42)	178.19 (159.07, 198.46)	189.96 (164.09, 220.85)	177.99 (160.23, 200.77)	197.30 (171.43, 229.34)	231.087	<0.001
TG (mg/dl)	116.81 (79.65, 176.99)	93.81 (69.91, 130.97)	128.32 (76.11, 224.78)	107.96 (79.65, 146.02)	162.39 (95.58, 253.98)	452.728	<0.001
HDL-C (mg/dl)	51.35 (43.63, 59.85)	50.19 (45.95, 54.83)	59.07 (39.00, 66.02)	50.19 (45.95, 54.83)	55.21 (39.77, 65.25)	81.570	<0.001
LDL-C (mg/dl)	102.32 (84.94, 120.08)	99.61 (86.49, 114.29)	100.77 (81.85, 122.78)	102.32 (87.84, 117.18)	105.41 (84.94, 129.34)	36.332	<0.001
Non-HDL-C (mg/dl)	133.59 (111.58, 157.14)	128.57 (108.88, 147.10)	135.52 (111.20, 162.55)	128.57 (109.65, 149.42)	143.24 (115.83, 173.26)	141.155	<0.001
RC (mg/dl)	29.34 (13.90, 43.63)	27.41 (13.42, 38.61)	32.05 (13.51, 49.03)	26.64 (12.74, 38.42)	32.82 (15.44, 51.74)	77.174	<0.001
TC/HDL-C	3.64 (3.06, 4.22)	3.59 (3.13, 4.00)	3.62 (2.90, 4.48)	3.61 (3.14, 4.00)	3.87 (3.06, 4.68)	57.117	<0.001
TG/HDL-C	0.99 (0.63, 1.63)	0.83 (0.60, 1.15)	1.11 (0.54, 2.15)	0.93 (0.68, 1.27)	1.44 (0.67, 2.49)	266.166	<0.001
LDL-C/HDL-C	2.02 (1.64, 2.39)	2.01 (1.69, 2.32)	1.94(1.48, 2.39)	2.05 (1.74, 2.34)	2.07 (1.62, 2.57)	26.279	<0.001
Taking lipid lowering drugs	19 (0.5)	0 (0.0)	3 (0.3)	0 (0.0)	16 (0.5)	31.729	<0.001

*All continuous variables are tested for normality (Shapiro-Wilk test and Kolmogorov–Smirnov test), and none of them obey normal distribution. Values are presented as median (interquartile range) or n (%)*.

*BMI, body mass index; TC, total cholesterol; TG, triglycerides; HDL-C, high-density lipoprotein cholesterol; LDL-C, low-density lipoprotein cholesterol; Non-HDL-C, non-high-density lipoprotein cholesterol; RC, remnant cholesterol*.

a *: For each quantitative variable, the P-value is obtained by the Mann-Whitney U test; for each categorical variable, the P-value is obtained through Pearson's χ^2^ test*.

### Association of Dyslipidemia and Hypertension With IS

As of July 27^th^, 2020, the median follow-up time was 10.76 years. There were 272 new IS with a prevalence density of 65.41 per 10,000 person-years during the follow-up period. The incidence density was higher among hypertensive patients, those with dyslipidemia, and both together, at 43.48 per 10,000 person-years, 91.52 per 10,000 person-years, and 92.28 per 10,000 person-years, respectively, compared to those with normal lipids and no hypertension. The corresponding HRs (95%) CIs are 1.603 (1.097–2.343), 1.025 (0.677–1.553) and 1.627 (1.129–2.343), respectively (Table 2).

**Table 2:** Association of dyslipidemia and hypertension with IS.

**Dyslipidemia**	**Hypertension**	**IS cases**	**Follow-up time (person-years)**	**Incidence density (/10,000 person-years)**	**HR(95%CI)^**a**^**	***P*** **^a^**
No	No	42	10,749.9	39.07	Ref	-
	Yes	78	8,522.4	91.52	1.603 (1.097–2.343)	0.015
Yes	No	48	11,040.22	43.48	1.025 (0.677–1.553)	0.907
	Yes	104	11,270.54	92.28	1.627 (1.129–2.343)	0.009

*IS, Ischemic stroke*.

a *: Adjusted for age, gender, BMI, cigarette smoking, alcohol consumption, T2DM at baseline, and use of lipid-lowering drugs*.

### Association Between Blood Lipids and the Risk of IS

Non-HDL-C ≥ 190 mg/dl was associated with an increased risk of IS in the whole population, but the correlation was not statistically significant after adjusting the covariates (Supplementary Table S1). The HR (95%CI) before and after adjustment were 1.529 (1.028–2.275) and 1.128 (0.751–1.694), respectively. The remaining lipid indices showed no statistically significant association with IS among the total population (Supplementary Table S1).

### Stratification Analysis of the Association Between Blood Lipids and the Risk of IS by Hypertension

In the hypertensive population, Non-HDL-C ≥ 190 mg/dl and LDL-C/HDL-C ≥ 2 were significantly associated with increased risk of IS, after adjusting for covariates, HRs (95%CIs) were 1.565 (1.007–2.429) and 1.414 (1.034–1.933), respectively. HDL-C ≥ 60 mg/dl was significantly associated with reduced risk of IS with adjusted HR (95%CI) of 0.665 (0.450–0.983). While in the non-hypertension population, no significant association of Non-HDL-C ≥ 190 mg/dl, LDL-C/HDL-C ≥ 2, and HDL-C ≥ 60 was detected with IS (*P* > 0.05).

Additionally, there was a significant association between TC/HDL-C ≥ 3.6 and the decreased risk of IS in the non-hypertension population, and the HR (95%CI) was 0.479 (0.307–0.750). However, a similar association was not observed in the hypertensive population.

Further analysis of heterogeneity test results indicated that the association of HDL-C ≥ 60 mg/dl, Non-HDL-C ≥ 190 mg/dl, TC/HDL-C ≥ 3.6, and TG/HDL-C ≥ 1 and IS are heterogeneous between hypertension and non-hypertension groups (*P*_heterogeneity_ < 0.05). See Table 3 for details.

**Table 3:** Hypertension status stratified analyses of abnormal lipid indices and the risk of IS.

**Lipid indices**	**Group**	**Non-hypertension**		**Hypertension**		${\text{P}}_{\text{heterogeneity}}^{\text{b}}$
		**HR (95% CI)^**a**^**	***P*** **^a^**	**HR (95% CI)^**a**^**	***P*** **^a^**	
TC	<240 mg/dl	reference	-	reference	-	
	≥240 mg/dl	0.445 (0.176–1.123)	0.086	1.224 (0.801–1.868)	0.350	0.052
TG	<200 mg/dl	reference	-	reference	-	
	≥200 mg/dl	0.714 (0.373–1.368)	0.310	1.254 (0.891–1.765)	0.194	0.133
HDL-C	40–60mg/dl	reference	-	reference	-	
	<40 mg/dl	0.884 (0.477–1.639)	0.695	1.157 (0.778–1.720)	0.471	0.472
	>60 mg/dl	1.411 (0.883–2.253)	0.150	0.665 (0.450–0.983)	0.041	0.016
LDL-C	<160 mg/dl	reference	-	reference	-	
	≥160 mg/dl	0.401 (0.056–2.880)	0.364	1.000 (0.542–1.847)	0.999	0.385
Non-HDL-C	<190 mg/dl	reference	-	reference	-	
	≥190 mg/dl	0.319 (0.098–1.036)	0.057	1.565 (1.007–2.429)	0.046	0.013
RC	<30 mg/dl	reference	-	reference	-	
	≥30 mg/dl	0.749 (0.488–1.148)	0.184	1.071 (0.795–1.442)	0.651	0.179
TC/HDL-C	<3.6	reference	-	reference	-	
	≥3.6	0.479 (0.307–0.750)	0.001	1.303 (0.955–1.778)	0.094	<0.001
TG/HDL-C	<1	reference	-	reference	-	
	≥1	0.698 (0.447–1.090)	0.114	1.259 (0.925–1.714)	0.143	0.033
LDL-C/HDL-C	<2	reference	-	reference	-	
	≥2	0.800 (0.523–1.223)	0.302	1.414 (1.034–1.933)	0.030	0.126

*TC, total cholesterol; TG, triglycerides; HDL-C, high-density lipoprotein cholesterol; LDL-C, low-density lipoprotein cholesterol; Non-HDL-C, non-high-density lipoprotein cholesterol; RC, remnant cholesterol*.

a *: Adjusted for age, gender, BMI, cigarette smoking, alcohol consumption, T2DM at baseline, and use of lipid-lowering drugs*.

^b^*: P_heterogeneity_ stands for the P value of the heterogeneity test*.

### Interaction Analysis of Abnormal Blood Lipid Indices and Hypertension for IS

We further analyzed the interaction between those indices which had heterogeneous associations with IS among different blood pressure statuses and hypertension. The results showed that HDL-C ≥ 60 mg/dl (vs HDL-C < 60 mg/dl), Non-HDL-C ≥ 190 mg/dl (vs. Non-HDL-C < 190 mg/dl), TC/HDL-C ≥ 3.6 (vs TC/HDL-C < 3.6), and TG/HDL-C ≥ 1 (vs. TG/HDL-C < 1) have additive and multiplicative interactions with hypertension. The RERIs (95% CIs) of the additive interaction are −0.93 (−1.882–0.044), 1.394 (0.38–2.407), 0.752 (0.354–1.151) and 0.575 (0.086–1.065), respectively. The HRs (95% CIs) of the multiplicative interaction terms were 0.498 (0.272–0.911), 4.218 (1.230–14.464), 2.423 (1.437–4.086) and 1.701 (1.016–2.848), respectively. In the analysis of the interaction between total dyslipidemia and hypertension, the association was not statistically significant. The RERI (95% CI) of the additive interaction was −0.002 (−0.635–0.632), while the HR (95% CI) of the multiplicative interaction term was 0.990 (0.595–1.645). See Table 4 for details.

**Table 4:** Analysis of the additive and multiplicative interaction of abnormal blood lipids and hypertension on IS.

**Interaction terms**	**Additive interaction**		**Multiplicative interaction**	
	**RERI (95% CI)^**a**^**	***p*** ^***a***^	**HR (95% CI)^**a**^**	***p*** ^***a***^
HDL-C (≥60 mg/dl) ^*^ HT	−0.963 (−1.882– −0.044)	0.040	0.498 (0.272–0.911)	0.024
Non-HDL-C (≥190 mg/dl) ^*^ HT	1.394 (0.38–2.407)	0.007	4.218 (1.230–14.464)	0.022
TC/HDL-C (≥3.6) ^*^ HT	0.752 (0.354–1.151)	0.001	2.423 (1.437–4.086)	0.001
TG/HDL-C (≥1) ^*^ HT	0.575 (0.086–1.065)	0.021	1.701 (1.016–2.848)	0.044
Dyslipidemia^*^HT	−0.002 (−0.635–0.632)	0.996	0.990 (0.595–1.645)	0.968

*TC, total cholesterol; TG, triglycerides; HDL-C, high-density lipoprotein cholesterol; Non-HDL-C, non-high-density lipoprotein cholesterol; RERI, relative excess risk due to interaction*.

a *: Adjusted for age, gender, BMI, cigarette smoking, alcohol consumption, T2DM at baseline, and use of lipid-lowering drugs*.

## Discussion

This study indicated that higher HDL-C levels will reduce the impact of hypertension on the risk of developing IS, while higher levels of Non-HDL-C, TC/HDL-C, and TG/HDL-C have a positive interaction effect with hypertension on the incidence of IS in Chinese adults.

Dyslipidemia is one of the major and modifiable risk factors for IS (23, 24). In China, the prevalence of adult dyslipidemia has increased significantly, from 18.6 in 2002 to 40.4% in 2012. Yet the awareness, treatment, and control rates of dyslipidemia are still low, at 31.0, 19.5, and 8.9%, respectively (12, 25, 26). Several studies have reported the association between cardiovascular disease and dyslipidemia, involving the conventional lipid indices such as TC, TG, LDL-C, HDL-C, and the lipid ratios such as TC/HDL-C, TG/HDL-C, and LDL-C/HDL-C. Two large prospective cohort studies both confirmed that high TC, TG, and LDL-C levels or low HDL-C levels increase the risk of CVD (27, 28). However, recent studies have also found a “U” or “J” shaped association between cholesterol levels (including TC, LDL-C, HDL-C) and CVD, with both high and low TC, LDL-C, and HDL-C levels contributing to adverse cardiovascular events (29–34). To comprehensively consider the effects of different lipid components on CVD risk, non-HDL-C, TC/HDL-C, TG/HDL-C, and LDL-C/HDL-C were proposed. Due to the ease of calculation of non-HDL-C and its strong predictive power for CVD, non-HDL-C was recommended as an independent risk factor for CVD (35, 36). Previous epidemiological studies have also demonstrated that higher levels of TC/HDL-C, TG/HDL-C, and LDL-C/HDL-C are associated with an increased risk of developing CVD.

In this study, stratification analysis showed that Non-HDL-C, LDL-C/HDL-C, and HDL-C were associated with IS in the hypertensive population and TC/HDL-C in the non-hypertensive population. These statistically significant indices are derived indices of HDL-C, which may indicate the important influence of HDL-C in the occurrence and development of IS. Besides, analyses of the association of dyslipidemia with IS demonstrated that, compared to LDL-C, non-HDL-C had a stronger relationship with IS risk in the hypertension stratified analysis. This finding may provide more evidence for non-HDL-C as a better predictor for atherogenesis risk than LDL-C in the hypertensive population. However, in the whole population analysis, none of the lipid indices showed a statistical association with IS. There are two main reasons. First, the cohort population and the number of patients with IS are relatively small, especially in performing subgroup analysis. So, it may be difficult to observe a statistically significant association, but the association trend can still be observed. Second, hypertension has a greater effect on the onset of IS, and the etiologic effect of dyslipidemia is relatively small. In the present study population, the prevalence of hypertension was as high as ~50%, so the effect of dyslipidemia may be masked.

Hypertension is another critical and controllable risk factor for IS. It can occur simultaneously with dyslipidemia and act synergistically to affect the risk of CVD according to previous research (21, 37). So, in this study, we analyze different results of the additive model and multiplicative models to evaluate the interaction between blood lipid indices and hypertension comprehensively. Our results suggested that HDL-C ≥ 60 mg/dl negatively affected IS in the hypertension group, from which we could infer that higher HDL-C levels may help reduce the impact of hypertension on IS risk. The underlying mechanism of this finding could be related to endothelial dysfunction in cerebral blood vessels. Atherosclerosis involving impairment of endothelial function and vascular contractility is an essential pathological basis for the development of IS (38). Hypertension-induced abnormalities in the cerebrovascular structure are known to play an important part in the pathogenesis of IS (39). Previous studies have shown that endothelium-mediated vasodilation is undermined in patients with essential hypertension (40–42). This abnormality is associated with attenuated endothelial Nitric Oxide (NO) activity and may be caused by selective abnormalities in NO synthesis (43). However, HDL particles could increase NO production by stimulating endothelial nitric oxide synthase (eNOS) activity and enhance endothelium- and NO-dependent relaxation in wild-type mice (44), which may help to explain, to some extent, the atheroprotective role of HDL-C in reducing the effect of hypertension on IS.

Non-HDL-C, TC/HDL-C, and TG/HDL-C, closely related to HDL-C, are all lipid indices derived from HDL-C but measure other lipids in the blood as well. Thus, these non-traditional lipid parameters may depict a more accurate and more comprehensive lipid profile than traditional ones. In the present study, non-HDL-C, TC/HDL-C, and TG/HDL-C had a positive interaction with hypertension for IS, implying that the abnormal imbalance of circulating lipids may amplify the effect of hypertension on IS. The pathophysiological mechanisms that could possibly explain the interaction between dyslipidemia and hypertension can be summarized as follows. First, LDL-C and hypercholesterolemia have been shown to make a difference in the interference for NO signaling activities resulting in the decrease of NO production and bioavailability, which consequently reduce endothelial vasolidaiton and enhance vasoconstructive activation. Therefore, dyslipidemia exacerbates the development of hypertensive status (45, 46). Moreover, subsequent vascular changes in function and structure caused by hypertension, such as altered hemodynamics at arterial bifurcations, as well as proinflammatory activities and oxidative stress, may worsen the harm produced by dyslipidemia (47). These can be supported by an animal study, which suggests that hypertension and hypercholesterolemia synergistically reduce endothelial function and increase oxidative stress in blood vessels in pigs, possibly exacerbating atherosclerosis due to dyslipidemia (48). In addition, previous research also indicated that the elevated LDL-C level indirectly increased the calcium influx into cells and stimulated vascular smooth muscle cell contraction (47).

With a follow-up of up to 10.75 years, this study systematically and prospectively investigated the interaction between lipid indices and hypertension on IS development. However, the following limitations exist. First, the sample size of this study was relatively small, which may reduce the efficacy of the test. Second, the study did not collect information on physical activity, whereas previous studies have shown that active physical activity reduces the risk of CVD morbidity and mortality by improving CVD risk factors. In addition, because the lipid data in this analysis were collected only at baseline, it was impossible to assess the effect of changes in lipid levels on CVD morbidity and mortality during follow-up. Therefore, a prospective cohort study with larger sample size, more comprehensive baseline information, and repeated lipid measurements during follow-up is urgently needed to validate the results of this study.

## Conclusion

This large prospective cohort study is the first for all we know to evaluate the combined effects of abnormal lipid parameters and hypertension on IS. The current results demonstrate that higher HDL-C levels may help reduce the impact of hypertension on IS risk and higher levels of non-HDL-C, TC/HDL-C, and TG/HDL-C, additively interacting with hypertension, increase the risk for developing IS. This study provides useful evidence for the combined effects of dyslipidemia and hypertension in predicting IS in Chinese adults. Therefore, the combination of non-traditional lipid indices and hypertension could aid in the screening process to identify high-risk populations before IS and may lead to more effective prevention of IS in clinical practice. Furthermore, because of the high comorbidity of dyslipidemia and hypertension and the interaction effect on IS, it is essential to implement lipids-control measures to prevent and treat IS in patients with hypertension in China. Targeting both dyslipidemia and hypertension in the clinical context could help build optimal therapeutic interventions for the prevention and management of IS.

## Data Availability Statement

The raw data supporting the conclusions of this article will be made available by the authors, without undue reservation.

## Ethics Statement

The studies involving human participants were reviewed and approved by Nanjing Medical University. The patients/participants provided their written informed consent to participate in this study.

## Author Contributions

LW: data analysis and manuscript preparation. JS and QZ: data acquisition and sorting. HX and CC: manuscript preparation. PW, XZ, YC, and ML: data acquisition. JD: data analysis. SY: study design and manuscript preparation. CS: study design, data interpretation, and manuscript preparation. All authors have read and approved the final manuscript.

## Funding

This work was supported by the National Natural Science Foundation of China (Grant No. 81872686, No.82173611, and No. 81573232), the Priority Academic Program for the Development of Jiangsu Higher Education Institutions (Public Health and Preventive Medicine), and the Flagship Major Development of Jiangsu Higher Education Institutions. The Funders had no role in the design and conduct of the study; collection, management, analysis, and interpretation of the data; preparation, review, or approval of the manuscript; and decision to submit the manuscript for publication.

## Conflict of Interest

The authors declare that the research was conducted in the absence of any commercial or financial relationships that could be construed as a potential conflict of interest.

## Publisher's Note

All claims expressed in this article are solely those of the authors and do not necessarily represent those of their affiliated organizations, or those of the publisher, the editors and the reviewers. Any product that may be evaluated in this article, or claim that may be made by its manufacturer, is not guaranteed or endorsed by the publisher.
