# Supplementary material for: Interaction Analysis of Abnormal Lipid Indices and Hypertension for Ischemic Stroke: A 10-Year Prospective Cohort Study
Source: Front Cardiovasc Med. 2022 Mar 11;9:819274. doi: 10.3389/fcvm.2022.819274 (PMC8962740; doi:10.3389/fcvm.2022.819274)
Supplement: Supplementary file 1 [file Table_1.docx]

**Table S1** Associations of abnormal lipid indices and the risk of IS among the whole population

| Lipids indices | groups | unadjusted | |  | adjusted | |
| --- | --- | --- | --- | --- | --- | --- |
|  |  | HR (95% CI) | *p* |  | HR (95% CI)^a^ | *p^a^* |
| TC | <240mg/dl | reference | - |  | reference | - |
|  | ≥240mg/dl | 1.309 (0.901-1.903) | 0.158 |  | 0.968 (0.661-1.416) | 0.865 |
| TG | <200mg/dl | reference | - |  | reference | - |
|  | ≥200mg/dl | 1.083 (0.810-1.447) | 0.591 |  | 1.096 (0.813-1.478) | 0.546 |
| HDL-C | 40-60mg/dl | reference | - |  | reference | - |
|  | <40mg/dl | 1.088 (0.783-1.512) | 0.616 |  | 1.082 (0.776-1.510) | 0.642 |
|  | >60mg/dl | 0.903 (0.674-1.211) | 0.497 |  | 0.877 (0.652-1.180) | 0.387 |
| LDL-C | <160mg/dl | reference | - |  | reference | - |
|  | ≥160mg/dl | 1.172 (0.657-2.091) | 0.590 |  | 0.878 (0.491-1.572) | 0.663 |
| Non-HDL-C | <190mg/dl | reference | - |  | reference | - |
|  | ≥190mg/dl | 1.529 (1.028-2.275) | 0.036 |  | 1.128 (0.751-1.694) | 0.563 |
| RC | <30mg/dl | reference | - |  | reference | - |
|  | ≥30mg/dl | 0.992 (0.782-1.259) | 0.950 |  | 0.953 (0.747-1.216) | 0.698 |
| TC/HDL-C | <3.6 | reference | - |  | reference | - |
|  | ≥3.6 | 1.023 (0.806-1.299) | 0.849 |  | 0.942 (0.734-1.208) | 0.637 |
| TG/HDL-C | <1 | reference | - |  | reference | - |
|  | ≥1 | 1.107 (0.873-1.405) | 0.402 |  | 1.051 (0.819-1.351) | 0.694 |
| LDL-C/HDL-C | <2 | reference | - |  | reference | - |
|  | ≥2 | 1.264 (0.994-1.609) | 0.056 |  | 1.166 (0.909-1.497) | 0.227 |
| Abbreviation: TC: total cholesterol; TG: triglycerides; HDL-C: high-density lipoprotein cholesterol; LDL-C: low-density lipoprotein cholesterol; Non-HDL-C: non-high-density lipoprotein cholesterol; RC: remnant cholesterol.  ^a^: Adjusted for age, gender, BMI, cigarette smoking, alcohol consumption, hypertension at baseline, T2DM at baseline, and use of lipid-lowering drugs. | | | | | | |
